# Outbreak of Human Infection with *Sarcocystis nesbitti*, Malaysia, 2012

**DOI:** 10.3201/eid1912.120530

**Published:** 2013-12

**Authors:** Sazaly AbuBakar, Boon-Teong Teoh, Sing-Sin Sam, Li-Yen Chang, Jefree Johari, Poh-Sim Hooi, Harvinder-Kaur Lakhbeer-Singh, Claire M. Italiano, Sharifah F. Syed Omar, Kum-Thong Wong, Norlisah Ramli, Chong-Tin Tan

**Affiliations:** University of Malaya, Kuala Lumpur, Malaysia (S. AbuBakar, B.-T. Teoh, S.-S. Sam, L.-Y. Chang, J. Johari, K.-T. Wong, N. Ramli);; University of Malaya Medical Centre, Kuala Lumpur (P.-S. Hooi, H.-K. Lakhbeer-Singh, C.M. Italiano, S.F. Syed Omar, C.-T. Tan)

**Keywords:** Sarcocystis nesbitti, parasites, protozoa, emerging infectious disease, human infection, fever, myalgia, myositis, Pangkor Island, Malaysia

## Abstract

An outbreak of fever associated with myalgia and myositis occurred in 2012 among 89 of 92 college students and teachers who visited Pangkor Island, Malaysia. The *Sarcocystis nesbitti* 18S rRNA gene and sarcocysts were obtained from muscle tissues of 2 students. Our findings indicate emergence of *S. nesbitti* infections in humans in Malaysia.

*Sarcocystis* spp. infections are emerging parasitic infections among travelers to potentially disease-endemic areas of Southeast Asia. More than 100 travelers acquired an acute, muscular, *Sarcocystis* spp. infection–like illness while traveling to and from Tioman Island, Malaysia, during 2011–2012 (*1*). Several cases were histologically confirmed by detection of intramuscular sarcocysts. Before these reports associated with travel to Tioman Island, <100 cases of intramuscular infection with *Sarcocystis* spp. had been reported (*2**–**4*) in humans. Earlier studies with tongue tissues obtained in an autopsy series suggested an infection prevalence of ≤21% among Malaysians (*5*). However, routine diagnostic examination of >1,500 limb muscle biopsy specimens in the past 20 years for various muscle diseases at the University of Malaya Medical Centre did not yield any sarcocyst-positive tissues (K.T. Wong, unpub. data). This finding suggests that human infection with *Sarcocystis* spp. is rare or that most of the infections are silent, mild and self-limited (*6*), or under-recognized.

There are >100 *Sarcocystis* spp. known and most have been isolated from muscle tissues of various intermediate hosts, including mammals, birds, and reptiles. *Sarcocystis* spp. are parasites with dual hosts to accommodate their dual life cycles. The sexual reproductive stage occurs in the definitive host, which appears to be relatively species constrained. During this stage, parasite activity is limited to the intestinal tract. In contrast, the asexual reproductive stage occurs in the intermediate host and appears to be relatively less species constrained. This stage occurs in the vascular endothelium and culminates in formation of mature muscle sarcocysts (*6*). However, *Sarcocystis* spp. infections in humans as the accidental intermediate host have been reported as intramuscular sarcocysts of unknown species (*7*).

## The Study

An outbreak investigation was undertaken after 89 symptomatic persons from Malaysia came to our institute after a college retreat during January 17–19, 2012, on Pangkor Island, Malaysia (4°13′52.35′′N, 100°32′44.55′′E). Ninety-two persons attended the retreat, which was held in a small hotel on the coast of the island; all outdoor activities were conducted on the beach or in the ocean. Eighty-nine symptomatic case-patients were identified with onset of fever (94%), myalgia (91%), headache (87%), and cough (40%) ≤26 days upon return. In persons who had a fever, the fever had a relapsing-remitting nature in 57% of patients.

Investigation by using magnetic resonance imaging (MRI) was prompted by development of visible swelling of the face in 9 patients and swelling of the calf muscles in 4 patients. Eight patients who had facial swelling and myalgia for 4–6 weeks underwent whole-body MRI by using the 1.5T Signa HDx MR System (GE Healthcare, Pittsburgh, PA, USA). All 8 patients showed changes in muscles of mastication, including superficial temporalis and deep temporalis, and in masseter muscles. Abnormalities were also observed in back muscles in 4 patients and in calf muscles in 2 patients. Muscle affected showed asymmetric high signal intensities on T2-weighted short T1 inversion recovery, consistent with inflammatory edema. A biopsy specimen was obtained from the temporalis muscle of 1 of these patients. Two leg muscle biopsy specimens were obtained from 2 other patients who reported specific muscle pain and had changes consistent with myositis by MRI. Mild myositis (inflammation) was observed in 3 muscle biopsy specimens examined.

Muscle tissues were ground with sterile glass beads by using a Precellys 24 homogenizer (Bertin Technologies, Montigny le Bretonneux, France) at 5,500 rpm for 30 s. Ten microliters of homogenates was inoculated into various cell cultures for virus isolation. Virus was not isolated from homogenates. RNA and DNA were also extracted from tissue homogenates by using the QIAamp Viral RNA Mini Kit and QIAamp DNA Mini Kit (QIAGEN, Hilden, Germany), respectively. PCR amplification for detection of infectious agents was performed. No specific amplification was obtained by using available primers for commonly detected viruses, including alphaviruses and other arboviruses. However, a *Sarcocystis* sp.18S rRNA gene was detected by using 5 primer pairs described (*8*).

Amplified DNA fragments were purified and sequenced by using the BigDye Terminator v3.1 Cycle Sequencing Kit on an automated capillary DNA sequencer 3730XL DNA Analyzer (Applied Biosystems, Foster City, CA, USA). Sequences were aligned with all available *Sarcocystis* spp. 18S rRNA sequences from GenBank. A neighbor-joining phylogenetic tree was constructed by using the maximum composite likelihood method as implemented in MEGA5 (www.megasoftware.net/).

**Figure 1:**
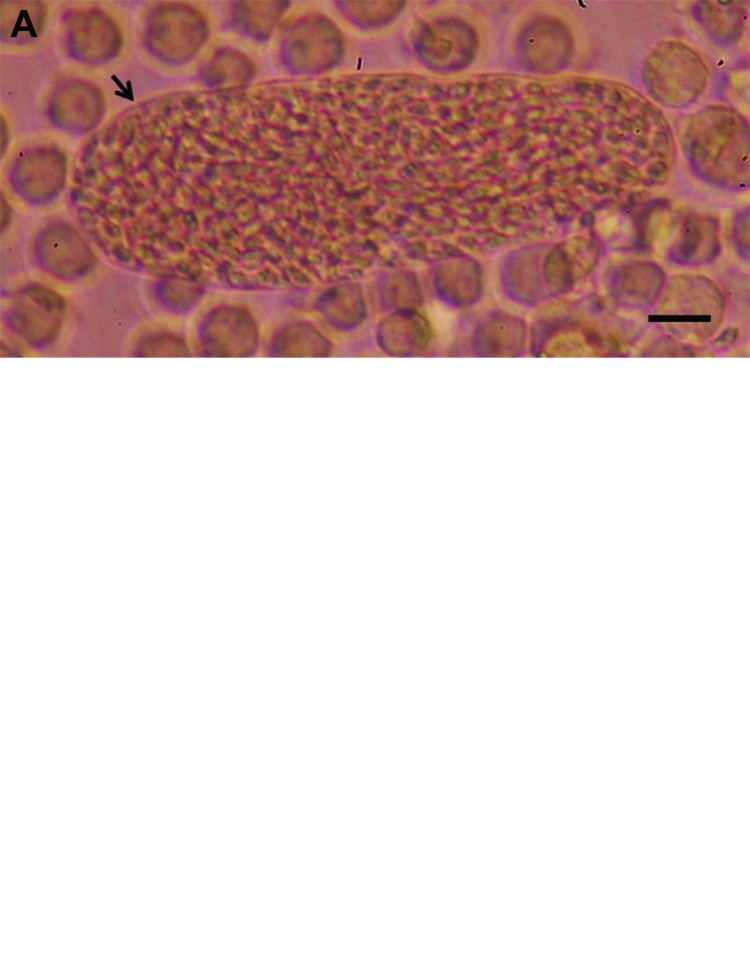
A) Sarcocysts isolated from persons infected with *Sarcocystis nesbetti*, Pangkor Island, Malaysia, 2012. Intact human sarcocyst (length 190 µm) with thin cyst wall (arrow) from homogenized temporalis tissue inoculated into a U937 monocytic cell culture (original magnification ×200, scale bar = 20 µm). B) Intramuscular sarcocyst enclosed by a thin smooth cyst wall (arrow) without any protrusions. Maximum cyst wall thickness is ≈0.5 µm (hematoxylin and eosin stained, original magnification ×40, scale bar = 10 µm).

Typical sarcocysts (length ≈190 µm) were observed in cell cultures inoculated with the muscle tissue homogenates of 1 of the patients (Figure 1, panel A) and directly in the muscle tissue of another patient (Figure 1, panel B). Attempts were made to culture sarcocysts from muscle tissue homogenates in U937 and THP-1 human monocytic cell lines but no propagation of bradyzoites was obtained. Nucleic acid amplification of the 2 tissue samples consistently showed DNA fragments with expected sizes of 329–1,208 bp. One tissue sample was from the temporalis muscle of 1 patient, and the other was from the leg muscle of another patient.

Both patients reported relapsing-remitting fever (3 episodes each), myalgia, and headache. Despite the presence of myositis, neither patient had increased serum creatinine phosphokinase levels but did have increased eosinophil counts of 1.0–2.6 × 10^9^ cells/L (reference range 0.02–0.50 × 10^9^ cells/L).

**Figure 2:**
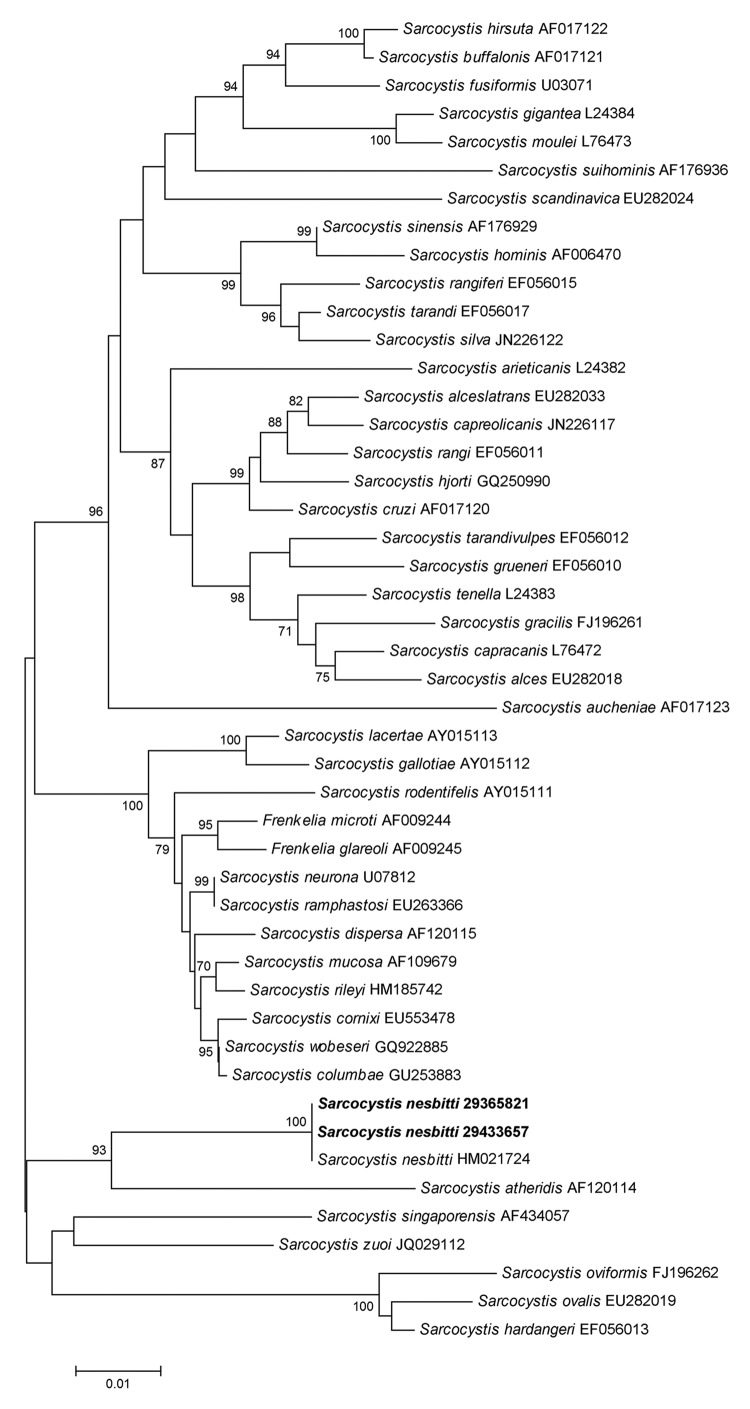
Neighbor-joining phylogenetic tree of *Sarcocystis* spp. 18S rRNA sequences. *Sarcocystis nesbitti* strains isolated in this study are indicated in **boldface**. Numbers at nodes indicate bootstrap values (%) for 1,000 replicates. Bootstrap values <70% are not shown. Scale bar indicates nucleotide substitutions per site.

DNA sequences obtained from 1,812-bp fragments were compared with fragments in GenBank by using BLAST (http://blast.ncbi.nlm.nih.gov/). Sequences matched 100% of those reported as *Sarcocystis nesbitti* (*9*). A phylogenetic tree constructed by using sequences *S. nesbitti* MY29365821 (GenBank accession no. HF544323) and *S. nesbitti* MY29433657 (accession no. HF544324) and those available in GenBank placed the 2 sequences in the clade with *S. nesbitti* and *S. atheridis* (*9*) (Figure 2).

## Conclusions

There have been several reports of *Sarcocystis* spp. infection in Malaysia and of tourists who had traveled to Malaysia (*1**,**2**,**5**,**10**–**13*). However, the *Sarcocystis* species was not identified in any of these reports. Only *S. hominis* and *S. suihominis* have been identified as the cause of human infections. In these instances, infections were believed to be asymptomatic, although minor self-limiting gastroenteritis was possible. Humans are definitive hosts for both species but only intestinal infections (no intramuscular sarcocysts) have been observed (*6*).

We report symptomatic *S. nesbitti* infection in humans. Predominant manifestations were fever (relapsing in ≈50% of patients), myalgia, headache, and cough. Although only 2 patients were confirmed to be acutely infected with *S. nesbitti*, it was likely that the remaining students and teachers in the group had the same infection because nearly all had similar signs and symptoms with onset of illness within days of each other. In addition, 9 patients had a distinctive facial myositis, but sarcocysts could not be verified in all of them because only 3 patients agreed to provide a muscle biopsy specimen. No other microorganisms were isolated from cell cultures of blood or other body fluids of patients.

*S. nesbitti* was reported by Mandour in 1969 in skeletal muscles of *Macaca mulatta* monkeys (*14*). Its presence in *M. fascicularis* monkeys, but not in humans, was reported in China by Yang et al. (*15*). Similar to infection in monkeys, it is likely that humans are also accidental intermediate hosts. Its definitive host is still unknown but earlier phylogenetic analysis suggests that snakes could be a probable definitive host (*9*). Our findings highlight the emergence of *S. nesbitti* infections in humans and suggest that these infections might be endemic to Malaysia.
